# Supplementary material for: A universal Urbach rule for disordered organic semiconductors
Source: Nat Commun. 2021 Jun 28;12:3988. doi: 10.1038/s41467-021-24202-9 (PMC8238995; doi:10.1038/s41467-021-24202-9)
Supplement: Supplementary file 1 — Supplementary Information [file 41467_2021_24202_MOESM1_ESM.pdf]

Supplementary Information

A Universal Urbach Rule for Organic Semiconductors

Christina Kaiser[1], Oskar J. Sandberg[1*], Nasim Zarrabi[1], Wei Li[1], Paul Meredith[1], Ardalan Armin[1*]

[1]Sustainable Advanced Materials (Sêr-SAM), Department of Physics, Swansea University, Singleton Park, Swansea SA2 8PP, United Kingdom

[*]Email: ardalan.armin@swansea.ac.uk; o.j.sandberg@swansea.ac.uk

Supplementary Note 1: Density of states

The energy difference between the ground state and excited state of a chromophore (single mode) is denoted by E_j'. In a system, containing a large number of chromophores (multimode), the density of states will follow a distribution with a maximum at the energy difference $E_{j,0}$. For a monoenergetic distribution of states we then have:

$$g_{\mathrm{DOS}}(E_j') = n_j \delta(E_j' - E_{j,0}), \tag{1}$$

where $g_{\mathrm{DOS}}(E_j')$ is the density of states (DOS) and n_{j} is the number density of sites with δ being Dirac Delta function. In disordered organic semiconductors, the energetic distribution of E_j' is often assumed to be Gaussian (centred around E_j), with a width (Gaussian energetic disorder) σ_s, taking the form

$$g_{\mathrm{DOS}}(E_j') = \frac{n_j}{\sqrt{2\pi\sigma^2}} \exp\left(-\frac{\left[E_j' - E_{j,0}\right]^2}{2{\sigma_s}^2}\right). \tag{2}$$

In contrast, in (banded) crystalline semiconductors the so-called tail states below the bands, caused by static disorder, are often modelled based upon an exponentially distributed density of states according to

$$g_{\mathrm{DOS}}(E_j') = \frac{n_j}{W} \exp\left(\frac{E_j' - E_{j,0}}{W}\right), \tag{3}$$

for $E_j' \leq E_{j,0}$, where W describes the width of the exponential distribution in analogy to σ_s of the Gaussian DOS.

Supplementary Note 2: Marcus theory

Rate constant. Marcus theory was originally derived to describe the electron transfer in electrochemical reactions. Herein, the energy of the ground state and excited state are approximated by parabolic potential energy surfaces that depend on the nuclear coordinates. Apart from describing the electron transport in disordered semiconductors, the Marcus theory, in the limit of weak coupling, has been successfully used in literature to describe optical transition rates. The rate constant between ground state and excited state can be expressed as

$$\kappa = \frac{2\pi}{\hbar} |v|^2 \frac{1}{\sqrt{4\pi\lambda_{j,0} kT}} \exp\left\{-\frac{\left(E_j' - E\right)^2}{4\lambda_{j,0} kT}\right\}, \tag{4}$$

where E is the photon energy, $|v|^2$ is the interstate coupling (or transfer integral) that is related to the transition dipole moment μ, and $\lambda_{j,0}$ is the reorganization energy that takes the polaronic effects into account.

Singlemode absorption coefficient. From the literature-known relationship between the absorption coefficient and rate constant,[1,2] the singlemode absorption coefficient from Marcus theory can be obtained as

$$\alpha_0(E, E_j') = \frac{f}{E\sqrt{4\pi k_B T \lambda_{j,0}}} \exp\left\{-\frac{\left(E_j' + \lambda_{j,0} - E\right)^2}{4\lambda_{j,0} kT}\right\}, \quad (5)$$

where f is the oscillator strength that includes the interstate coupling and other energy-independent parameters.

Multimode absorption coefficient with Gaussian DOS. After integrating over all Gaussian distributed energy states (DOS from Supplementary Equation (2)) via

$$\alpha(E) = \int_0^\infty \alpha_0(E, E_j') g(E_j')\, dE_j' \approx \int_{-\infty}^\infty \alpha_0(E, E_j') g(E_j')\, dE_j', \quad (6)$$

the multimode absorption coefficient can be written as

$$\alpha(E) = \frac{f_j}{E\sqrt{4\pi k_B T \lambda_j}} \exp\left\{-\frac{\left(E_j + \lambda_j - E\right)^2}{4\lambda_j' kT}\right\}. \quad (7)$$

where $f_j = n_j f$. The Gaussian spectral shape of the single mode expression from eq. S5 is reobtained in Supplementary Equation (7), however, with the parameters E_j' and $\lambda_{j,0}$ from the single mode absorption now replaced by

$$E_j = E_{j,0} - \frac{{\sigma_s}^2}{2kT}, \quad (8)$$

$$\lambda_j = \lambda_{j,0} + \frac{{\sigma_s}^2}{2kT}. \quad (9)$$

Note that the result for a monoenergetic DOS, Supplementary Equation (1), is obtained by setting $\sigma_s = 0$. For $\sigma_s \neq 0$, the multimode absorption coefficient is redshifted with respect to the singlemode absorption coefficient depending on σ_s. This additional linewidth broadening contribution is sometimes also referred to as the inhomogeneous broadening. The origin of linewidth broadness hence can be twofold. Recent studies have shown evidence that the linewidth broadening is mainly due to $E_{j,0}$ and $\lambda_{j,0}$, which stem from the coupling of vibrational and electronic states described by Marcus theory.[3,4] Supplementary Equation (7) is often applied to the experimental absorption coefficient or external quantum efficiency (EQE) in the spectral range of charge transfer (CT) absorption to extract the material-specific parameters E_j and λ_j, which are then called E_{CT} and λ_{CT} ($j = \text{CT}$). By increasing the sensitivity limit of the EQE measurement to 10^{-8} %,[5] it was recently shown[6] that the EQE in the spectral range of mid-gap trap states follows a Gaussian spectral line shape. The material-specific parameters

E_{t} and λ_{t} (Supplementary Equation (7) with $j = \mathrm{t}$) can be extracted from a fit with Supplementary Equation (7) to the experimental EQE spectrum to parametrize the mid-gap trap state absorption.

Multimode absorption coefficient with Exponential DOS. Under the assumption of an exponential tail distribution of energy states and a Marcus derived expression for the optical rate constant, the corresponding multimode absorption coefficient for tail state absorption (i.e. $E_j' < E_{j,0}$) is obtained from

$$\alpha(E) = \int_{-\infty}^{E_{j,0}} \alpha_0(E, E_j') g_{\mathrm{DOS}}(E_j')\, dE_j', \tag{10}$$

and thus

$$\alpha(E) = \frac{f_j}{2EW} \exp\left(\frac{E - (E_{j,0} + \lambda_{j,0} - \frac{\lambda_j kT}{W})}{W} \right) \times \left[\mathrm{erf}\left(\frac{E_{j,0} + \lambda_{j,0} - E - \frac{2\lambda_{j,0} kT}{W}}{\sqrt{4\lambda_{j,0} kT}} \right) + 1 \right]. \tag{11}$$

where $\mathrm{erf}(x)$ is the error function. Importantly, unlike for the integration over the Gaussian distributed states, exponential tail states are only defined for energies below the gap. Hence, the upper integration bound is given by $E_{j,0}$.

For photon energies $E \ll E_{j,0}$, the factor comprising the error functions converges to 1 and $\alpha(E)$ reduces to

$$\alpha(E) = \frac{f_j}{EW} \exp\left(\frac{E - E_j^*}{W} \right), \tag{12}$$

where $E_j^* = E_{j,0} + \lambda_{j,0} - \frac{\lambda_{j,0} kT}{W}$. In the spectral range, where absorption of organic photodiodes can be detected experimentally, Supplementary Equation (12) is dominated by the exponent and hence $\alpha(E)$ follows an Urbach decay, where the width of the DOS is the Urbach energy.

Supplementary Note 3: Miller Abrahams type rate

Rate constant. The Miller-Abrahams (MA) rate constant is commonly employed to describe the charge-transfer rate from site m to n in disordered semiconductors. This rate is usually of the form:

$$\kappa_{mn} = \tilde{v}_0 \times \begin{cases} \exp\left(-\frac{\Delta E_{mn}}{kT}\right) & : \Delta E_{mn} \geq 0 \\ 1 & : \Delta E_{mn} < 0 \end{cases} \tag{13}$$

where and $\tilde{v}_0$ is a prefactor that is related to the electronic coupling between the sites and depends on the inverse localization length, the distance between the localized states and the phonon frequency. Here, ΔE_{mn} is the energy difference between the two sites. The exponential term is a Boltzmann factor

describing the thermal activation of the hopping process. Note that two cases are distinguished: (i) for $\Delta E_{mn} \geq 0$, the rate is thermally activated; and (ii) for $\Delta E_{mn} < 0$, the rate is not impeded by the additional term.

As is shown below, under certain conditions Marcus theory can be extended with an energy dependence which may be approximated by a MA-type energy dependence. Similar to Marcus theory, the MA formalism can then be used to express an absorption rate constant. From this perspective, the absorption rate can be expressed as a function of E_j' and the photon energy E according to

$$\kappa \propto A \exp\left(-\frac{E_j' - E}{kT}\right). \quad (14)$$

for $E < E_j'$, while $\kappa \propto A$ for $E > E_j'$. As for Marcus theory, E_j' describes the energy required for an optical transition from the ground state to the excited state. For $E_j' = E$, the maximum rate is obtained. In analogy to the oscillator strength form Marcus theory, A is assumed to be only weakly dependent on the photon energy.

Singlemode absorption coefficient. Based on the transfer rate described by Supplementary Equation (14), we expect for $E_j' > E$

$$\alpha_0(E, E_j') = \alpha_{\text{sat}} \exp\left(-\frac{E_j' - E}{kT}\right), \quad (15)$$

where α_{sat} determines the maximum absorption coefficient in the spectral range where $E_j' < E$. α_{sat} further contains a $1/E$-dependence which has negligible effect at energies below the gap and above the experimental limit ($E > 0.5$ eV).

Multimode absorption coefficient with Gaussian DOS. Considering a Gaussian DOS and the single mode absorption coefficient from Supplementary Equation (15), the multimode absorption coefficient is calculated via

$$\alpha(E) = \frac{\alpha_{\text{sat}}}{\sqrt{2\pi\sigma_s^2}}\left[\int_{-\infty}^{E} \exp\left(-\frac{[E_j' - E_{j,0}]^2}{2\sigma_s^2}\right) dE_j' + \int_{E}^{\infty} \exp\left(\frac{E - E_j'}{kT}\right) \exp\left(-\frac{[E_j' - E_{j,0}]^2}{2\sigma_s^2}\right) dE_j'\right] \quad (16)$$

The integration over all sites with the energy E_j' is split into two parts: (i) $-\infty < E_j' < E$, where the optical transition is not impeded by the Boltzmann term (hence $\alpha_0 = \alpha_{\text{sat}}$) and (ii) for $E < E_j' < \infty$ where $\alpha_0 < \alpha_{\text{sat}}$. Note that the lower integration bound for (i) was taken to be $-\infty$ instead of 0 (this is valid for $E_{j,0} \gg kT$). After solving the integral, one obtains

$$\alpha(E) = \frac{\alpha_{\text{sat}}}{2}\left\{\exp\left(\frac{E - E_{j,0} + \frac{\sigma_s^2}{2kT}}{kT}\right)\left[1 - \text{erf}\left(\frac{E - E_{j,0} + \frac{\sigma_s^2}{kT}}{\sigma_s\sqrt{2}}\right)\right] + \text{erf}\left(\frac{E - E_{j,0}}{\sigma_s\sqrt{2}}\right) + 1\right\}. \quad (17)$$

Noting that $E_j = E_{j,0} - \frac{{\sigma_s}^2}{2kT}$ and that the error functions of Supplementary Equation (17) approach -1 for $E \ll E_j$, we find

$$\alpha(E) \approx \alpha_{\text{sat}} \exp\left(\frac{E - E_j}{kT}\right). \tag{18}$$

The energy dependence of $\alpha(E)$ is dominated by the exponential term in the relevant spectral range of sub-gap absorption and therefore $[d\ln(\alpha(E))/dE]^{-1} = kT$. For $E \gg E_j$, on the other hand, both error functions in Supplementary Equation (17) approach 1 and $\alpha(E) \to \alpha_{\text{sat}}$.

Multimode absorption coefficient with Exponential DOS. Considering an exponential DOS and Supplementary Equation (16) for the single mode absorption, the multimode absorption coefficient associated with tail state absorption is calculated according to Supplementary Equation (5). One finds

$$\alpha(E) = \alpha_{\text{sat}} \int_{-\infty}^{E} \exp\left(\frac{E_j' - E_{j,0}}{W}\right) dE_j' + \int_{E}^{E_{j,0}} \exp\left(\frac{E - E_j'}{kT}\right) \exp\left(\frac{E_j' - E_{j,0}}{W}\right) dE_j' \tag{19}$$

Similar to Supplementary Equation (17), the integration over all sites with the energy E_j' is split into two parts: (i) $-\infty < E_j' < E$, where the optical transition is not impeded by the Boltzmann term (hence $\alpha_0 = \alpha_{\text{sat}}$) and (ii) for $E < E_j' < E_{j,0}$ where $\alpha_0 < \alpha_{\text{sat}}$. Again, for an exponential DOS, it is assumed that there are no tail states for $E_j' > E_{j,0}$ and hence the upper bound is $E_{j,0}$ (instead of ∞ as for the Gaussian DOS). Solving the integral of Supplementary Equation (19) leads to

$$\alpha(E) = \alpha_{\text{sat}} \exp\left(\frac{E - E_{j,0}}{W}\right) \left\{ \frac{1 - \exp\left(\frac{E - E_{j,0}}{kT} - \frac{E - E_{j,0}}{W}\right)}{\frac{W}{kT} - 1} + 1 \right\}. \tag{20}$$

From Supplementary Equation (20), it is clear that $[d\ln(\alpha(E))/dE]^{-1}$ will be dominated by W, i.e. the width of the exponential distribution, as expected.

Supplementary Note 4: Understanding MA type rates in terms of generalized Marcus theory

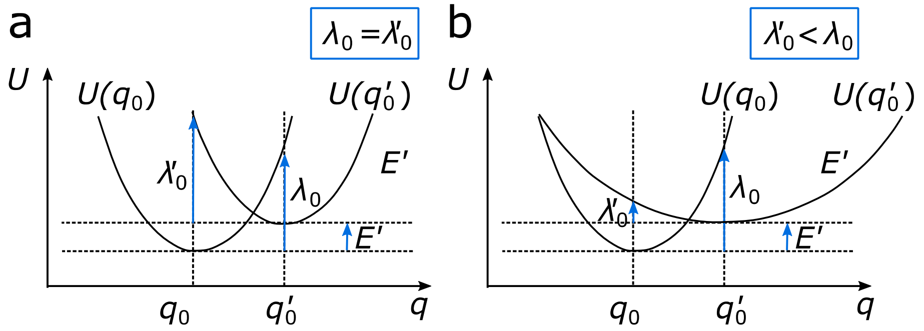

Supplementary Figure 1. Profile of the potential energy surfaces (PESs) of the ground state and the excited state, $U(q_0)$ and $U(q'_0)$, respectively. **a,** Equal curvature of the PESs lead to equal reorganization energies λ_0 and λ'_0 for the ground state and the excited state, respectively. **b,** Broadening of one PES with respect to the other leads to unequal reorganization energies.

In Marcus theory, the curvature of the potential energy surfaces (PES) describes the vibrational frequency of the modes and is directly related to the reorganization energy. Supplementary Fig. 1 illustrates the PES as a function of the nuclear coordinate q, where $U(q_0)$ and $U(q'_0)$ denote the ground state and the excited state, respectively. Two cases can be distinguished: a) for the standard Marcus theory, the curvatures of the PESs are equal and so are the reorganization energies of the ground state λ_0 and the excited states λ'_0. b) In the general case, the curvatures of the PESs are different resulting in different reorganization energies for the ground state and the excited state.

Case b) can be viewed as an extension to the standard Marcus theory with the generalized Marcus transfer rate now reading [7,8]

$$\kappa = \frac{2\pi}{\hbar} \frac{|v|^2(\tilde{k}_+ + \tilde{k}_-)}{\sqrt{4\left[\lambda'_0 + \left(1 - \frac{\lambda'_0}{\lambda_0}\right)(E' - E)\right]\pi kT}}. \tag{21}$$

Here, E' is the energy difference between the ground state and excited state. For $E < E' + \frac{\lambda_0 \lambda'_0}{(\lambda_0 - \lambda'_0)}$,

$$\tilde{k}_+ = \exp\left(-\frac{\lambda_0}{kT} \left[\frac{E' - E + \lambda'_0}{\lambda'_0 + \sqrt{{\lambda'_0}^2 + (\lambda_0 - \lambda'_0)(E' - E + \lambda'_0)}} \right]^2 \right) \tag{22}$$

$$\tilde{k}_{-} = \exp\left(-\frac{\lambda_0}{kT}\left[\frac{E' - E + \lambda_0'}{\lambda_0' - \sqrt{\lambda_0'^2 + (\lambda_0 - \lambda_0')(E' - E + \lambda_0')}}\right]^2\right) \quad (23)$$

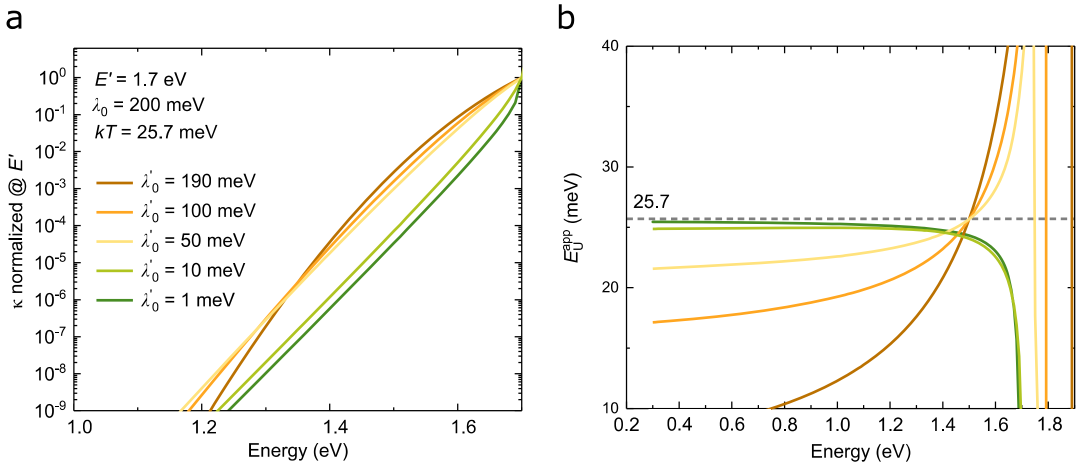

Supplementary Figure 2. a, Generalized Marcus transfer rate simulated for different λ_0' at room temperature assuming $\lambda_0 = 200$ meV and $E' = 1.7$ eV. κ approaching a Gaussian spectral line-shape for $\lambda_0'/\lambda_0 \to 1$ and an exponential line-shape for $\lambda_0'/\lambda_0 \to 0$. **b,** Apparent Urbach energy $E_{\mathrm{U}}^{\mathrm{app}}$ approaching kT for $\lambda_0'/\lambda_0 \to 0$.

Note that the rate reduces back to the standard Marcus charge-transfer rate (i.e. Supplementary Equation (4)) for $\lambda_0' \to \lambda_0$, as expected. This is demonstrated in Supplementary Fig. 2, where the full expression of κ from Supplementary Equation (21) is simulated for different ratios of λ_0'/λ_0. Supplementary Fig. 2a shows that the spectral lineshape of κ resembles a Gaussian function for λ_0'/λ_0 close to 1, while for $\lambda_0'/\lambda_0 \to 0$, it converges to an exponential function with an inverse logarithmic slope of kT. As a result, the apparent Urbach energy $E_{\mathrm{U}}^{\mathrm{app}}$ approaches kT for $\lambda_0'/\lambda_0 \to 0$, as illustrated in Supplementary Fig. 2b.

In the case of distribution for E' (e.g. Gaussian or exponential DOS), we need to simplify the generalized Marcus rate since a direct integration of Supplementary Equation (21) for a given DOS is in general analytically intractable. However, for $\lambda_0 > \lambda_0'$, the transfer rate at low photon energies, corresponding to the absorption tail, can be approximated by

$$\kappa \approx \frac{2\pi}{\hbar} \frac{|v|^2}{\sqrt{\left[\lambda_0' + \left(1 - \frac{\lambda_0'}{\lambda_0}\right)(E' - E)\right]\pi kT}} \exp\left(-\frac{[E' - E + \lambda_0']}{kT} \frac{\lambda_0}{(\lambda_0 - \lambda_0')}\right) \quad (24)$$

If $\lambda_0 \gg \lambda_0'$ (i.e. a more localized ground state and a more diffuse exited state), the dominant photon energy dependence of the absorption tail is of the form

$$\kappa \propto \exp\left(\frac{E - E'}{kT}\right) \tag{25}$$

becoming governed by a simple Boltzmann factor. Under such conditions, we may approximate the generalized Marcus rate with a simple MA-type expression.

**D:A blend**	E_{CT} (in eV)	λ_{CT} (in meV)	f_{CT} (in eV^2)	E_{t} (in eV)	λ_{t} (in meV)	f_{t} (in eV^2)
BQR:$PC_{70}BM$	1.42	233	9.1e-4	0.68	866	1.8e-6
PCDTBT:$PC_{70}BM$	1.48	350	5.4e-3	0.74	560	1.2e-7
PBDB-T:$PC_{70}BM$	1.40	355	3.0e-3	0.70	610	6.3e-7
PTB7-Th:ITIC	1.43	451	4.5e-2	0.71	947	1.9e-6
PBDB-T:ITIC	1.50	287	1.9e-3	0.87	458	2.6e-7
PBDB-T:IT-4F	1.42	461	2.0e-3	--	--	--
PM6:Y6	--	--	--	0.86	280	2.8e-8
PM6:ITIC	--	--	--	0.86	464	8.3e-7
PBDB-T:EH-IDTBR	--	--	--	0.86	583	1.5e-7

Supplementary Table 1. Parameters extracted from Gaussian fits to the experimental sub-gap EQE in the spectral range of CT absorption and mid-gap state absorption spectra for 9 different D:A blends. Empty cells indicate the absence of spectral features related to CT or trap state absorption.

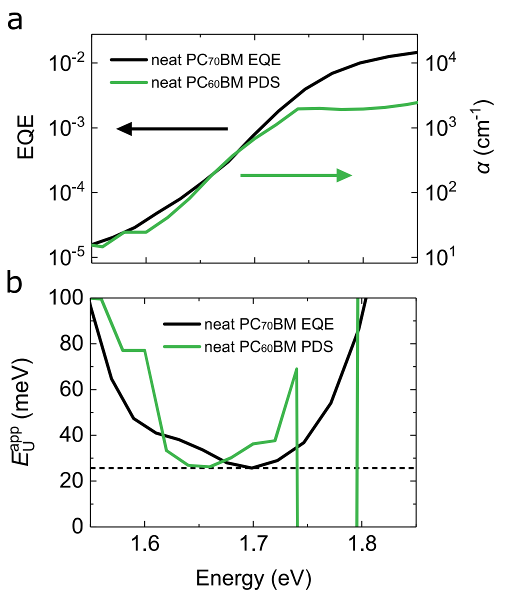

Supplementary Figure 3. a, Sub-gap EQE of a $PC_{70}BM$ solar cell (black line) and the absorption coefficient (α; green line) of a neat $PC_{60}BM$ film measured via photothermal deflection spectroscopy (PDS) are compared. The PDS data was taken from the literature.[9] **b,** The corresponding $E_{\mathrm{U}}^{\mathrm{app}}$ spectra calculated from the EQE of a $PC_{70}BM$ solar cell (black line) and from the α of a neat $PC_{60}BM$ film (green line) are shown. The EQE and PDS derived $E_{\mathrm{U}}^{\mathrm{app}}$ spectra show $E_{\mathrm{U}}^{\mathrm{app}} \approx kT$ at similar energies at around 1.70 eV and 1.65 eV, respectively, confirming that the spectral line-shape of EQE can be used as an approximation for α in the sub-gap energy range.

Supplementary Note 7: Thickness dependence of the EQE tail

Optical interference (or cavity) effects modify the EQE of a solar cell, which can be regarded as a low finesse cavity with a fully reflective top electrode (for example Ag) and a semi-transparent bottom electrode (often ITO). The resonance wavelength λ_{res} depends on the effective thickness t_{cav} of the cavity with $\lambda_{\mathrm{res}} = 2nt_{\mathrm{cav}}j^{-1}$, with j being a natural number and n is the refractive index. Supplementary Fig. 4a and 4b demonstrate the thickness dependence of the photovoltaic EQE above and below gap for PM6:ITIC and PM6:Y6 respectively. Note that the effective bandgap for both blends corresponds to the energy gap of the neat acceptor and hence the interference effects will strongly depend on the refractive index of the acceptor.

Moreover, the effect of imbalanced charge collection in "thick" organic semiconductor junctions ($t_{\mathrm{cav}} > 500$ nm) is visible as a decrease in the absolute EQE in the visible part of the EQE spectrum of PM6:ITIC as reported earlier for other D:A blends.[10] While the sub-gap slope above the spectral range seems to be unaffected on a logarithmic scale, the detailed analysis via the inverse slope of the

logarithmic EQE ($E_{\mathrm{U}}^{\mathrm{app}}$) reveals thickness-dependent resonance modes. Around the steepest point of the sub-gap EQE, $E_{\mathrm{U}}^{\mathrm{app}}$ deviates from the thermal energy kT by no more than 3 meV for PM6:ITIC and by maximally 6 meV for PM6:Y6. Such deviations are expected to increase with higher dispersion in the refractive index and extinction coefficient.

The effect of optical interference can be simulated using the well-established transfer-matrix model, which requires knowledge of the optical constants of the solar cell stack of interest. As the experimental absorption coefficient cannot be determined with sufficient sensitivity, we assume a perfectly exponential sub-gap slope with $E_{\mathrm{U}}^{\mathrm{app}} = kT$ for $E < 1.55$ eV. For $E > 1.55$ eV, the experimental above-gap absorption coefficient of a 200 nm thick PM6:ITIC film on silicon is used and its refractive index as determined from spectroscopic ellipsometry over the entire wavelength range. This fictional absorption coefficient normalized to its maximum in the above-gap spectral range is shown in Supplementary Fig. 5a. In the absence of any spectral dependence of the internal quantum efficiency, the absorptance calculated by the transfer-matrix model equals the EQE of the device. By changing the layer thickness of PM6:ITIC in the simulation, the spectral line-shape of the absorptance changes as demonstrated by the spectrum of $E_{\mathrm{U}}^{\mathrm{app}}$ in Supplementary Fig. 5b. In the experiment, $E_{\mathrm{U}}^{\mathrm{app}} \approx kT$ is observed between 1.6 and 1.3 eV. In this spectral range, the simulated $E_{\mathrm{U}}^{\mathrm{app}}$ shows deviations of +1 to -2 meV around kT, while discontinuities around 1.55 eV are due to the manual stitching of the experimental and fictional sub-gap absorption coefficient. As shown earlier, the fluctuation in the experimental $E_{\mathrm{U}}^{\mathrm{app}}$ for PM6:ITIC devices lies in between +3 to -3 meV and are therefore very close to the simulated deviations.

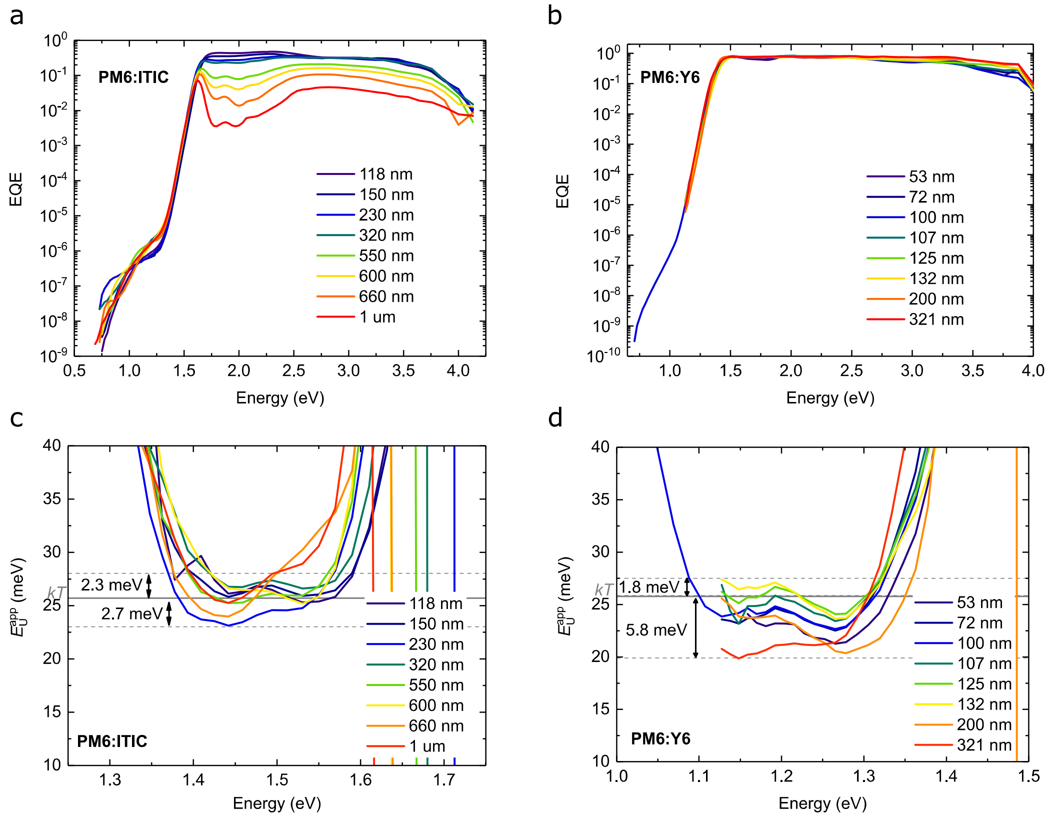

Supplementary Figure 4. Nearly exponential EQE tails observed for the PM6:ITIC (**a,**) and PM6:Y6 (**b,**) BHJs of different thicknesses in the spectral range below the gap and above the trap state absorption (1.4 eV $< E <$ 1.6 eV for PM6:ITIC and 1.1 eV $< E <$ 1.3 eV for PM6:Y6). In this spectral range, $E_{\mathrm{U}}^{\mathrm{app}}$ is close to kT for all thicknesses for both PM6:ITIC (**c,**) and PM6:Y6 (**d,**) while small deviations from kT can be attributed to weak optical cavity effects.

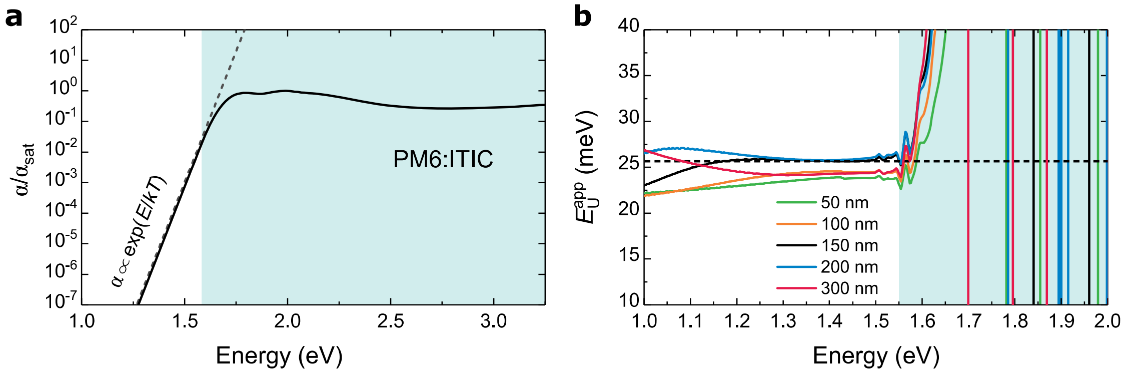

Supplementary Figure 5. a, Absorption coefficient (α) used in transfer-matrix simulations to calculate the absorptance of an active layer (device architecture ITO (100 nm)/ZnO (30 nm)/active layer/MoO_3 (8 nm)/Ag (100 nm)). **b**, Sub-gap E_U^{app} for 5 different active layer thicknesses (50 to 300 nm) showing energy and thickness dependence within +1/-2 meV range around kT for 1.3 eV $< E <$ 1.6 eV. Optical cavity effects, included in the transfer-matrix simulation, are thus expected to cause spectral dependence of E_U^{app} in a real device.

Supplementary Note 8: Temperature dependent EQE measurements and simulations

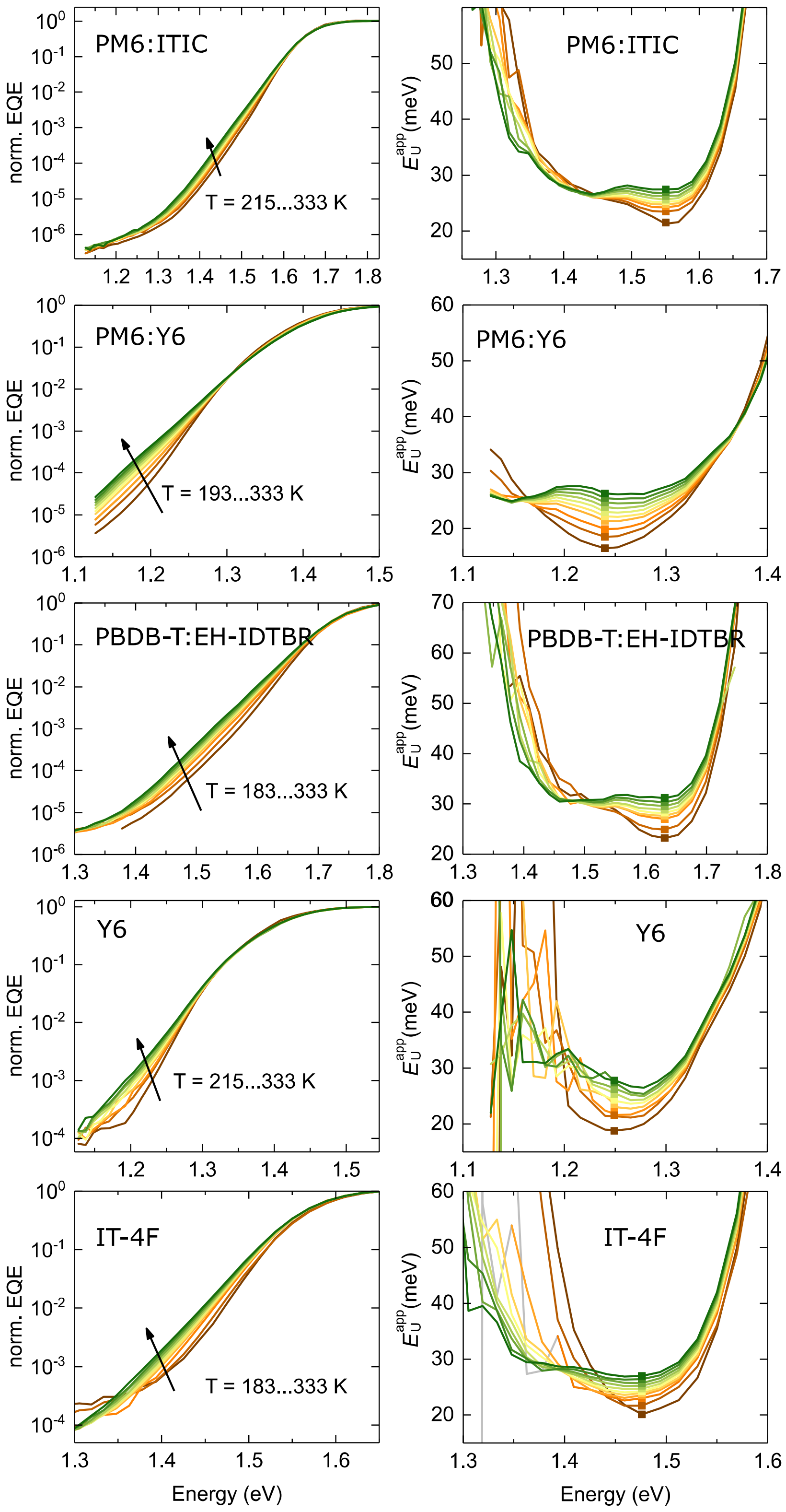

Supplementary Figure 6. Temperature-dependent EQE and respective apparent Urbach energy spectra of neat and blend material systems.

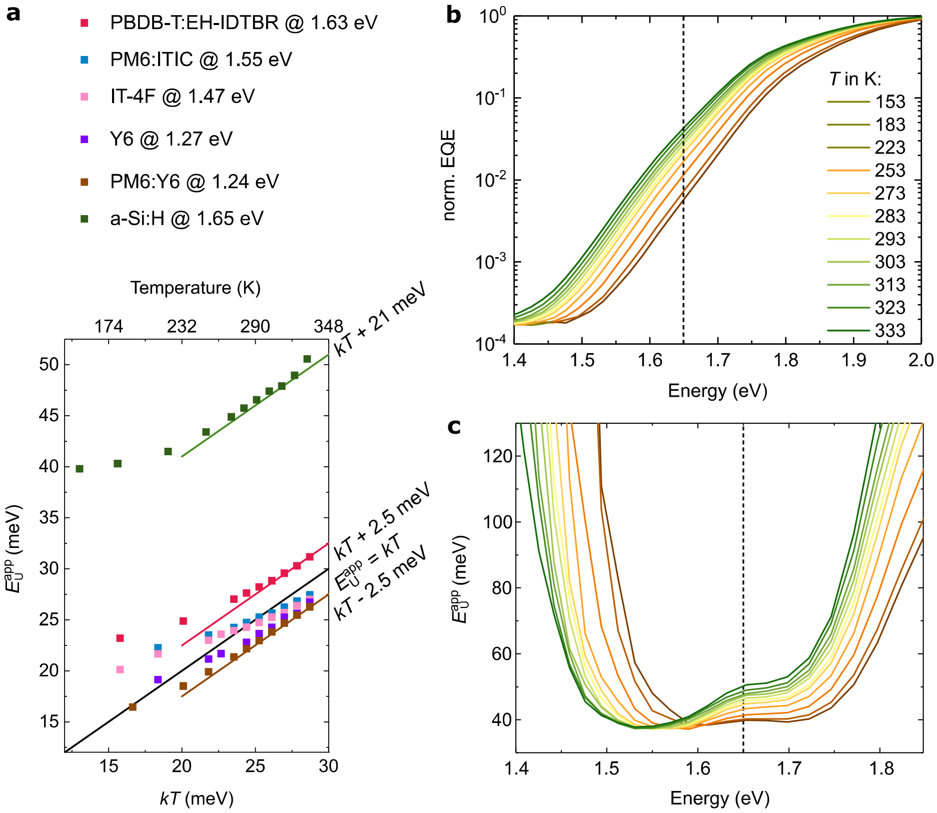

Supplementary Figure 7. a, $E_{\mathrm{U}}^{\mathrm{app}}$ at constant energy for a commercial a-Si:H solar cell and organic BHJs and neat layer solar cells as a function of temperature. **b,** Normalized sub-gap EQE of a commercial a-Si:H solar cell at different temperatures. **c,** Apparent Urbach energy $E_{\mathrm{U}}^{\mathrm{app}}$ as calculated from the sub-gap EQE. The dashed line indicates the energy at which energy $E_{\mathrm{U}}^{\mathrm{app}}$ was evaluated.

At room temperature, the excitonic absorption coefficient tails of low-offset material systems can be approximated with an Urbach energy given by $E_{\mathrm{U}}^{\mathrm{app}} \approx kT$. To prove the generality of this statement, EQE spectra were recorded at different temperatures between 183 to 333 K for three low-offset BHJs solar cells and two neat (acceptor-only) material systems. Supplementary Fig. 6 shows EQE spectra normalized to the above-gap absorption and the respective $E_{\mathrm{U}}^{\mathrm{app}}$. To study $E_{\mathrm{U}}^{\mathrm{app}}$ as a function of temperature, $E_{\mathrm{U}}^{\mathrm{app}}$ is evaluated at a constant energy below the presumed optical gap and above the spectral range of deep trap state absorption, as indicated by solid markers or a dashed for each material system. The slope of the EQE at this energy decreases as the temperature increases, i.e. $E_{\mathrm{U}}^{\mathrm{app}}$ increases with T. Supplementary Fig. 7a illustrates $E_{\mathrm{U}}^{\mathrm{app}}$ as a function of kT (and T). For all tested material systems at temperatures above room temperature, $E_{\mathrm{U}}^{\mathrm{app}}$ converges to a linear function that equals $E_{\mathrm{U}}^{\mathrm{app}} = kT$ + constant. The constant, i.e. the intercept of the linear function, is no more than $\pm$2.5 meV and can be attributed to optical interference as shown earlier.

Next, temperature-dependent EQE spectra were recorded for a commercial a-Si:H thin-film solar cell, as shown in Supplementary Fig. 7b. Note that due to the junction thickness of 200 to 400 nm, interference effects are expected[11] to affect $E_{\mathrm{U}}^{\mathrm{app}}$. Reported Urbach energies are usually 40 meV and above depending on the degree of hydrogenation[12] and the energy range considered[13,14]. In literature, E_{U} of a-Si:H was previously described as $E_{\mathrm{U}}(T) = E_{\mathrm{U,D}}(T) + E_{\mathrm{U,S}}$, where $E_{\mathrm{U,S}}$ is the T-independent contribution and $E_{\mathrm{U,D}}(T)$ increases with T. $E_{\mathrm{U,S}}$ is hereby the result of imperfections and structural disorder in the band structure of Si, while the direct excitation of defect states is usually reported at energies below 1.5 eV.[12,15] According to the sub-gap $E_{\mathrm{U}}^{\mathrm{app}}$ of a-Si:H shown in Supplementary Fig. 7c, the spectral broadening close to the bandgap shows stronger temperature dependence in comparison to organic semiconductors. At the same time, $E_{\mathrm{U}}^{\mathrm{app}}$ reaches a plateau at 40 meV independent of temperature which is 2-3 orders of magnitude below the absorption band edge. For a consistent analysis in comparison to organic BHJs, $E_{\mathrm{U}}^{\mathrm{app}}$ is evaluated at a constant energy above the direct defect absorption, here corresponding to 1.65 eV. As displayed in Supplementary Fig. 7a, $E_{\mathrm{U}}^{\mathrm{app}}$ saturates for low temperatures to roughly 40 meV, while the thermal activation at higher temperatures at 1.65 eV seems to follow $E_{\mathrm{U}}^{\mathrm{app}}(1.65\ \mathrm{eV}) = kT + 21\ \mathrm{meV}$, which is marked by a line as guide to the eye.

To model the experimental observation of $E_{\mathrm{U}}^{\mathrm{app}} \approx kT$ in the spectral range of sub-gap LE absorption, it is reasonable to assume a thermally activated rate constant similar to a Miller-Abrahams (MA) type charge-transfer. This is also supported by the generalized Marcus theory assuming unequal potentials for the ground state and excited state as was shown previously. For the macroscopic description of the absorption in a material, one needs to account for the distribution of states which, in the case of organic semiconductors, is often considered to be Gaussian (see Supplementary Equation (17)) and sometimes exponential (see Supplementary Equation (20)). Hereby, the spectral line-shape of the distribution is determined by $E_{j,0}$, i.e. the energy corresponding to the peak value of the distribution, and W or σ_s, corresponding to the broadening of the exponential or Gaussian distribution, respectively. To simulate the sub-gap absorption coefficients, W and σ_s are both assumed to be 70 meV, being within the range of previously reported values for static energetic disorder.[16–19] Moreover, $E_{j,0} = 1.7$ eV which roughly equals the energy gap of a typical BHJ solar cells.

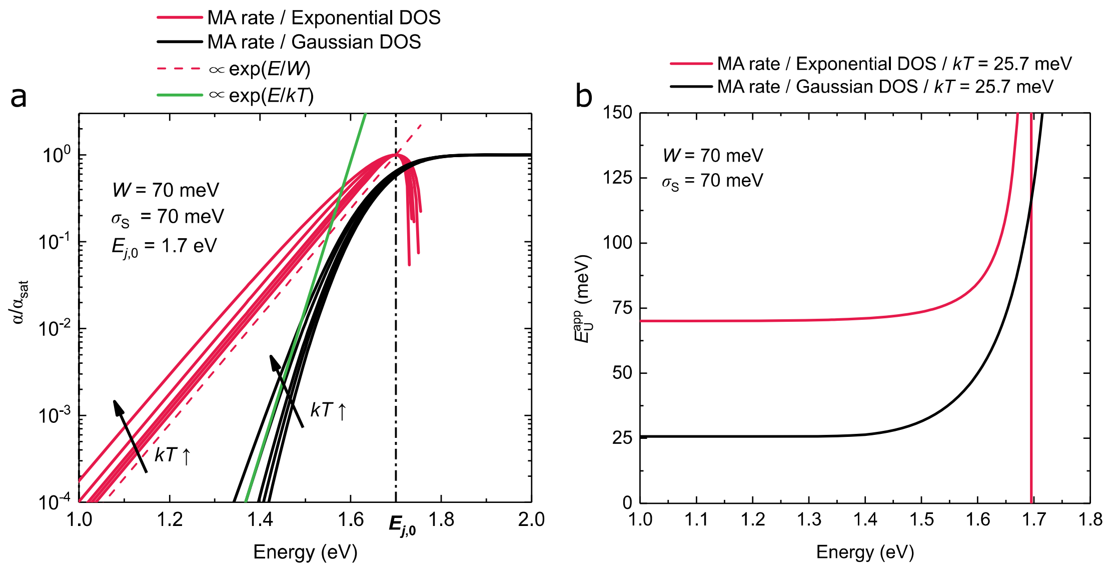

Supplementary Figure 8. Calculated absorption coefficient based on MA-type rate constants normalized to α_{sat}. **a,** α derived for exponential DOS (red lines; normalized at $E = E_{j,0}$) and for Gaussian DOS (black lines) at kT between 13 - 30 meV. Green line: Guideline for the eye illustrating a function that is proportional to $\exp(E/kT)$. **b,** α derived from the MA-type rate constant using an exponential DOS showing that $E_{\text{U}}^{\text{app}} = W$ in the limit $E \ll E_{j,0}$.

Supplementary Fig. 8 shows the calculated absorption coefficient α normalized to α_{sat} based on Supplementary Equation (17) and (20) for an exponential (red lines) and Gaussian (black lines) DOS at different temperatures. Note that for the exponential tail DOS, α has a maximum at $E_{\text{j,0}}$ and is not defined for $E > E_{j,0}$. In the case of a Gaussian DOS, the spectral range around $E_{\text{j,0}}$ is dominated by an error function which converges to a constant α_{sat}. Supplementary Fig. 8a illustrates that the temperature-dependent sub-gap slope with $E_{\text{U}}^{\text{app}} = kT$ can only be reproduced by a MA-type rate function derived for a Gaussian DOS at $E \ll E_{j,0}$. In contrast, an exponential DOS is independent of the temperature resulting in $E_{\text{U}}^{\text{app}} = W$ (cf. Supplementary Fig. 8b) and can therefore not explain the experimental findings. To conclude, only the result for a Gaussian DOS is consistent with the experimental results.

In the next step, the influence of σ_s on the sub-gap slope is examined in Supplementary Fig. 9 by considering the dominant contributions to Supplementary Equation (17) in the high-energy regime (highlighted in blue) and the low-energy regime (highlighted in purple) separately. Importantly, the spectral shape of Supplementary Equation (17) in the high-energy limit is determined by σ_s, which redshifts the effective bandgap by ${\sigma_s}^2/2kT$, causing an overall broadening of the sub-gap absorption coefficient. Therefore, the upper limit of the spectral range where $E_{\text{U}}^{\text{app}} = kT$ is determined by σ_s. The purple part of the equation dominates the spectral range where $E \ll E_{j,0}$ and is independent of σ_s.

Measured EQE spectra show that the trap state absorption is typically 6 orders of magnitude weaker than above-gap absorption and poses a lower limit to the spectral range where $E_{\mathrm{U}}^{\mathrm{app}} = kT$. Apart from the experimental limitation of measuring small EQE signals, it is hence possible that for some organic semiconductors $E_{\mathrm{U}}^{\mathrm{app}} = kT$ cannot be observed at room temperature due to large σ_{s}.

a

$$\frac{\alpha(E)}{\alpha_{\mathrm{sat}}} = \frac{1}{2}\left\{\exp\left(\frac{E - E_{j,0} + \frac{\sigma_{\mathrm{s}}^2}{2kT}}{kT}\right)\left[1 - \mathrm{erf}\left(\frac{\frac{\sigma_{\mathrm{s}}^2}{kT} + E - E_{j,0}}{\sigma_{\mathrm{s}}\sqrt{2}}\right)\right] + \mathrm{erf}\left(\frac{E - E_{j,0}}{\sigma_{\mathrm{s}}\sqrt{2}}\right) + 1\right\}$$

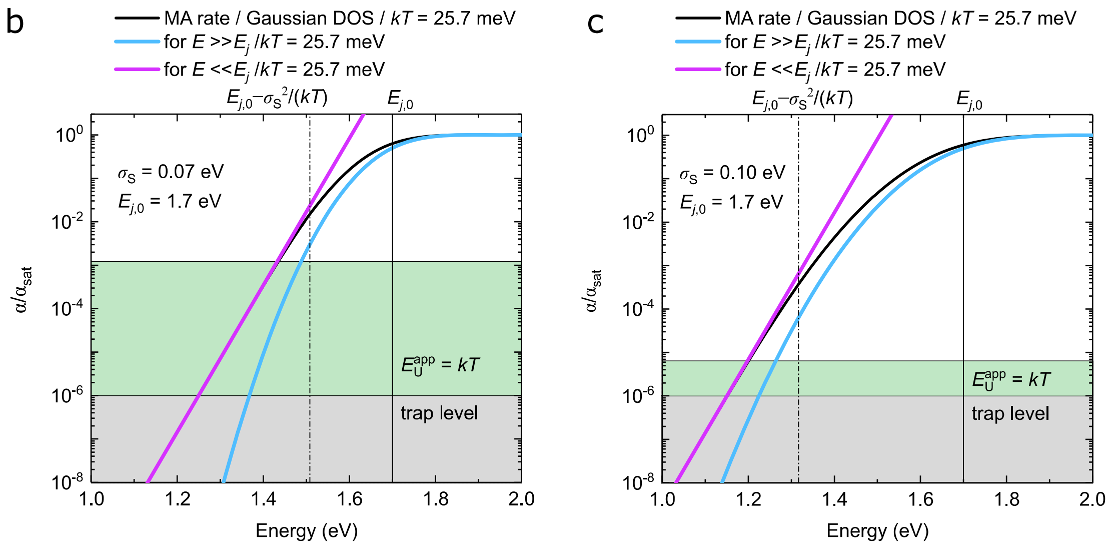

Supplementary Figure 9. a, Absorption coefficient derived for MA-type rate and Gaussian DOS. Purple and green colours indicate α in the limit of $E \ll E_{j,0}$ and $E \gg E_{j,0}$ respectively. Normalized α for a Gaussian DOS characterized by $E_{j,0} = 1.7$ eV and **b,** $\sigma_{\mathrm{s}} = 0.07$ eV and **c,** $\sigma_{\mathrm{s}} = 0.1$ eV at room temperature. For $E \ll E_{j,0}$, α converges to an exponential function with a slope described by $E_{\mathrm{U}}^{\mathrm{app}} = kT$. For $E \gg E_{j,0}$, α converges to 1 if normalized to α_{sat}. The larger σ_{s}, the more sensitive the EQE measurement must be to show that $E_{\mathrm{U}}^{\mathrm{app}} = kT$ at low energies. Roughly 6 – 7 orders of magnitude below α_{sat}, deep trap state absorption typically dominates the spectrum as shown experimentally. Hence, $E_{\mathrm{U}}^{\mathrm{app}} = kT$ cannot observed experimentally for $\sigma_{\mathrm{s}} \geq 0.1$ eV.

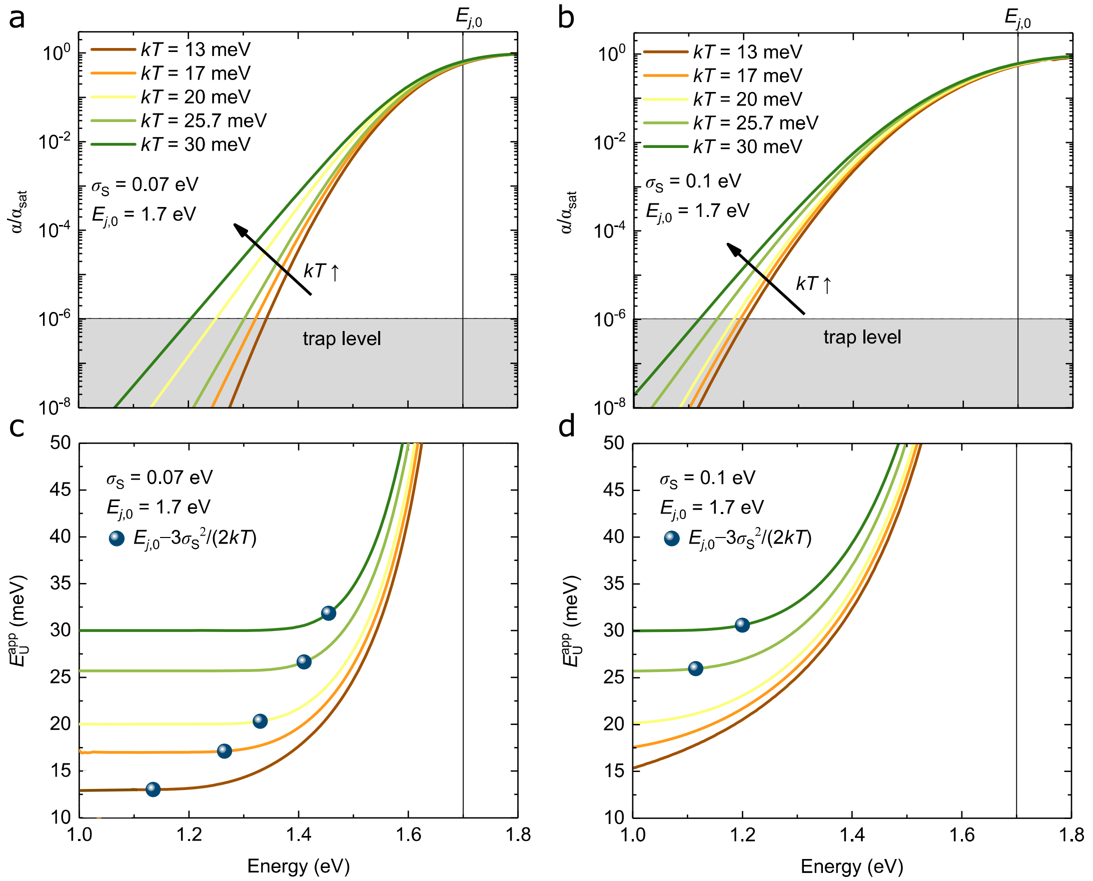

Supplementary Figure 10. Simulation of the absorption coefficient derived for MA-type rate and Gaussian DOS at different temperatures for **a,** $\sigma_s = 0.07$ eV and **b,** $\sigma_s = 0.1$ eV and the respective E_U^{app} for **c,** $\sigma_s = 0.07$ eV and **d,** $\sigma_s = 0.1$ eV. By increasing σ_s and/or decreasing T, the energy at which E_U^{app} reaches the plateau where $E_U^{app} = kT$ is redshifted. For comparison, the point $E = E_{j,0} - 3\sigma_s^2/(2kT)$ has been indicated as marked by the blue spheres.

Supplementary Fig. 10 shows the corresponding absorption coefficients from Supplementary Fig. 9, but for different temperatures (at $\sigma_s = 70$ meV and $\sigma_s = 100$ meV). The comparison of Supplementary Fig. 10a and 10b shows that the spectral range where $E_U^{app} = kT$ increases with temperature but decreases for higher σ_s. This may partly also explain the deviation from $E_U^{app} = kT$ observed for PBDB-T:EH-IDTBR and PM6:Y6 at low temperatures. In the simulation, a rather high degree of static disorder, e.g. $\sigma_s = 100$ meV, leads to the plateau of $E_U^{app} = kT$ only being reached at $kT > 25.7$ meV and $E < 1.2$ eV. As shown in the experimental EQE spectra, this energy regime is typically dominated by mid-gap trap absorption or the low energy tail of the CT absorption at low temperatures. Therefore, in the framework of this theory, it is unlikely to observe $E_U^{app} = kT$ for a material with a density of LE states characterized by $\sigma_s = 100$ meV when $E_{j,0} = 1.7$ eV.

To further validate our model (Supplementary Equation (17)), σ_s and $E_{j,0}$ are extracted as fit parameters for neat IT-4F and Y6 from experimental EQE spectra recorded at different temperatures as shown in Supplementary Fig. 11. Herein, the same energy range was chosen for all temperatures when fitting for one material system to exclude

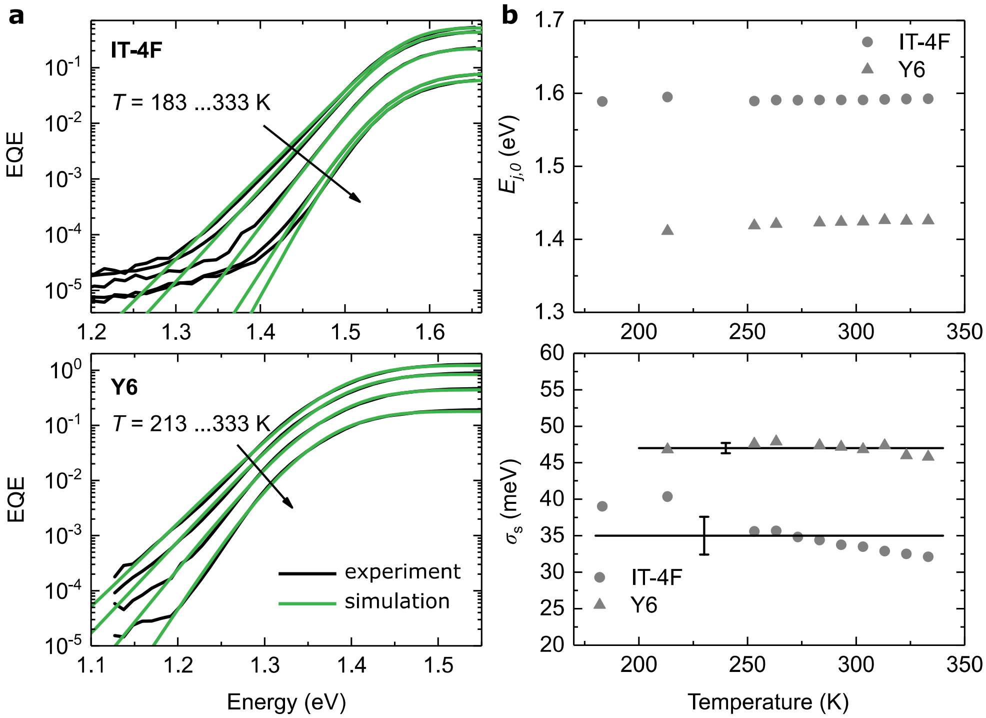

Supplementary Figure 11. a, Black lines: Sub-gap EQE of a neat IT-4F solar cells at 183, 213, 263, 303 and 333 K, and sub-gap EQE of a neat Y6 solar cell at 213, 263, 303 and 333 K. Green lines: Simulated sub-gap EQE at different temperatures. **b,** Static disorder σ_s and the exciton band gap energy $E_{j,0}$ obtain as fit parameters of the simulated sub-gap absorption coefficient.

low energy contributions from the trap state absorption. An advantage of neat material systems is that charge-transfer absorption is absent. The results for IT-4F and Y6 are shown for a few temperatures in Supplementary Fig. 11a, while the fit parameters σ_s and $E_{j,0}$ are displayed for all temperatures in Supplementary Fig. 11b. $E_{j,0}$ shows negligible temperature dependence for both materials as well as σ_s for Y6. The standard deviations of the experimentally-extracted σ_s over all temperatures are indicated by error bars in Supplementary Fig. 11b. For IT-4F, σ_s decreases with temperature resulting in a standard deviation of 2.7 meV. The origin of this apparent trend is beyond the scope of this study.

Finally, σ_s and $E_{j,0}$ are extracted from EQE measurements at 303 K for the three exemplary low-offset D:A systems PM6:Y6, PM6:ITIC and PBDB-T:EH-IDTBR. For above gap, the EQE spectra are normalized to 1.

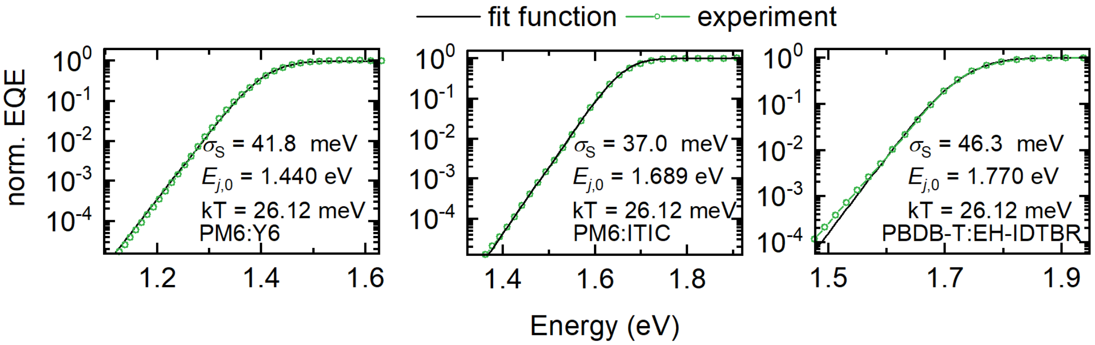

Supplementary Figure 12. Green lines: Normalized experimental EQE spectra measured at 303 K for the low-offset system D:A systems PM6:Y6, PM6:ITIC and PBDB-T:EH-IDTBR. Black lines: The corresponding simulated sub-gap EQE spectra. Static disorder σ_s and the exciton band gap energy $E_{j,0}$ are obtained as fit parameters.

Supplementary References

1. Nelson, J. *The Physics of Solar Cells*. (Imperial College Press, London, 2003).

2. May, V. & Kühn, O. *Charge and Energy Transfer Dynamics in Molecular Systems*. (Wiley-VCH Verlag GmbH & Co. KGaA, Berlin, 2011).

3. Tvingstedt, K., Benduhn, J. & Vandewal, K. Temperature dependence of the spectral line-width of charge-transfer state emission in organic solar cells; static vs. dynamic disorder. *Mater. Horizons* **7**, 1888–1900 (2020).

4. Panhans, M. *et al.* Molecular vibrations reduce the maximum achievable photovoltage in organic solar cells. *Nat. Commun.* 1–10 (2020).

5. Zeiske, S., Kaiser, C., Meredith, P. & Armin, A. Sensitivity of Sub-Bandgap External Quantum Eifficiency Measurements of Solar Cells under Electrical and Light Bias. *ACS Photonics* **7**, 256–264 (2019).

6. Zarrabi, N. *et al.* Charge-generating mid-gap trap states define the thermodynamic limit of organic photovoltaic devices. *Nat. Commun.* **11**, 5567 (2020).

7. Casado-pascual, J., Morillo, M., Goychuk, I., Hänggi, P. & Morillo, M. The role of different reorganization energies within the Zusman theory of electron transfer The role of different reorganization energies within the Zusman theory of electron transfer. *J. Chem. Phys.* **118**, 291 (2003).

8. Tang, J. Electron-transfer reactions involving non-linear spin-boson interactions. *Chem. Phys.* **188**, 143–160 (1994).

9. Buchaca-Domingo, E. *et al.* Direct correlation of charge transfer absorption with molecular donor:acceptor interfacial area via photothermal deflection spectroscopy. *J. Am. Chem. Soc.* **137**, 5256–5259 (2015).

10. Lin, Q., Armin, A., Burn, P. L. & Meredith, P. Filterless narrowband visible photodetectors. *Nat. Photonics* **9**, 687–694 (2015).

11. Leblanc, F. Related content Accurate Determination of the Urbach Energy of a- Si : H Thin Films by Correction for the Interference Effect. *Jpn. J. Appl. Phys.* **33**, L1755–L1758 (1994).

12. Cody, G. D., Tiedje, T., Abeles, B., Brooks, B. & Goldstein, Y. Disorder and the

optical-absorption edge of hydrogenated amorphous silicon. *Phys. Rev. Lett.* **47**, 1480–1483 (1981).

13. Searle, T. M. & Jackson, W. A. Static versus electron-phonon disorder in amorphous Si:H and its alloys. *Philos. Mag. B* **60**, 237–255 (1989).

14. Cody, G. D. Urbach edge of crystalline and amorphous silicon: a personal review. *J. Non. Cryst. Solids* **141**, 3–15 (1992).

15. Smith, Z. E. *et al.* Photothermal and photoconductive determination of surface and bulk defect densities in amorphous silicon films. *Appl. Phys. Lett.* **50**, 1521–1523 (1987).

16. Hood, S., Zarrabi, N., Meredith, P., Kassal, I. & Armin, A. Measuring Energetic Disorder in Organic Semiconductors Using the Photogenerated Charge-Separation Efficiency. *J. Phys. Chem. Lett.* **10**, 3863–3870 (2019).

17. Burke, T. M., Sweetnam, S., Vandewal, K. & Mcgehee, M. D. Beyond Langevin Recombination : How Equilibrium Between Free Carriers and Charge Transfer States Determines the Open-Circuit Voltage of Organic Solar Cells. *Adv. Energy Mater.* **1500123**, 1–12 (2015).

18. Arkhipov, V. I., Emelianova, E. V., Tak, Y. H. & Bässler, H. Charge injection into light-emitting diodes: Theory and experiment. *J. Appl. Phys.* **84**, 848–856 (1998).

19. Noriega, R. *et al.* A general relationship between disorder, aggregation and charge transport in conjugated polymers. *Nat. Mater.* **12**, 1038–1044 (2013).
